# Evaluating the Frequency of Esophageal Motility Disorders in Patients With Upper Gastrointestinal Symptoms Based on the Chicago Classification Version 3.0

**DOI:** 10.7759/cureus.100609

**Published:** 2026-01-02

**Authors:** Yorinari Ochiai, Yasutaka Kuribayashi, Akihiro Yamada, Hiroyuki Odagiri, Yugo Suzuki, Junnosuke Hayasaka, Satoshi Yamashita, Akira Matsui, Daisuke Kikuchi, Shu Hoteya

**Affiliations:** 1 Gastroenterology, Toranomon Hospital, Tokyo, JPN; 2 Okinaka Memorial Institute for Medical Research, Toranomon Hospital, Tokyo, JPN

**Keywords:** chicago classification version 3.0, dysphagia, esophageal motility disorders, high-resolution manometry, patients with upper gastrointestinal stmptoms

## Abstract

Introduction: Patients with upper gastrointestinal (GI) symptoms may have esophageal motility disorders (EMDs). Esophageal high-resolution manometry (HRM) is often performed when no obvious abnormalities are observed on esophagogastroduodenoscopy (EGD). However, few reports from Japan have evaluated the relationship between symptoms and EMDs in a large number of patients from a single institution. This study aimed to elucidate the frequency of EMDs and the relationship between patient symptoms and EMDs by evaluating HRM findings based on the Chicago Classification version 3.0 (CC v3.0).

Methods: A total of 258 patients with upper GI symptoms (258 examinations) who had no organic abnormalities on EGD and underwent their first HRM at our institution between July 2013 and May 2022 were enrolled. We retrospectively analyzed the proportion of EMDs in patients with upper GI symptoms and examined the relationship between upper GI symptoms and EMDs.

Results: The mean age of the patients was 57 ± 15 years, and 42.4% were male. The most common symptom was dysphagia, observed in 101 patients (39.1%), followed by heartburn in 51 patients (19.8%). The proportion of EMDs based on CC v3.0 was 45.3% (117 of 258 patients) among all patients with upper GI symptoms and 65.3% (66 of 101 patients) among those with dysphagia; this difference was statistically significant (p < 0.001). Additionally, various types of EMDs were observed in patients with dysphagia including 45.5% (30 of 66 patients) of patients diagnosed as achalasia type I-III.

Conclusion: 45.3% of the patients with upper GI symptoms were diagnosed with EMDs on HRM based on CC v3.0. Patients presenting with dysphagia were more likely to have EMDs. HRM may be a useful diagnostic modality for patients with upper GI symptoms who have no organic abnormalities on EGD. In particular, HRM should be considered in daily clinical practice for patients with dysphagia, given the potential presence of EMDs, including achalasia.

## Introduction

Esophageal motility disorders (EMDs) are motility dysfunction of the esophagus that can cause symptoms such as dysphagia and chest pain, despite the absence of obvious abnormalities on esophagogastroduodenoscopy (EGD). Esophageal high-resolution manometry (HRM) is used to accurately define esophageal motor function, define abnormal motor function, and delineate a treatment plan based on motor abnormalities [1]. HRM is widely performed and considered the gold standard for diagnosing EMDs. The Chicago Classification, the first diagnostic system, was initially proposed in 2009 [2] and has been continually updated [3-5].

However, some patients experience intolerance to HRM due to factors such as gagging, coughing, anxiety and visceral hypersensitivity [6]. Understanding the symptoms associated with EMDs is crucial for determining the appropriate indication for HRM. Previous studies have reported dysphagia as the only symptom with a high likelihood ratio and positive predictive value for identifying major motility disorders [7,8]. However, EMDs encompass various symptoms, including chest pain and heartburn [9]. There are limited reports focusing on the relationship between EMDs and symptoms in a large cohort of patients from a single institution in Japan. Additionally, previous studies have shown that 15% of patients with EMDs were missed on endoscopy within one year prior to the manometric diagnosis [10]. This makes the relationship between symptoms and EMDs particularly important in daily practice, especially in symptomatic patients without endoscopic findings. The Chicago Classification version 4.0 [5] has been introduced recently; therefore, the Chicago Classification version 3.0 (CC v3.0) [4] continues to include a large number of cases.

Therefore, this study aimed to elucidate the proportion of EMDs and their relationship with patient symptoms, such as dysphagia and heartburn, by evaluating HRM using CC v3.0 in a large cohort of patients with upper gastrointestinal (GI) symptoms at a single institution in Japan.

## Materials and methods

Study design

This was a single-center retrospective study.

Patients

**Figure 1:**
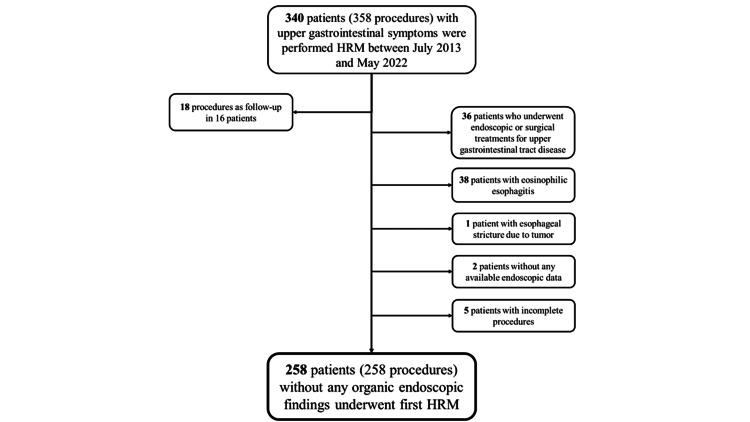
Flow diagram of this study. HRM, high-resolution manometry.

A total of 340 patients (358 examinations) with upper GI symptoms underwent HRM at our institution between July 2013 and May 2022. All patients underwent EGD prior to the HRM to exclude organic abnormalities that could cause symptoms, including upper GI neoplasms. Thirty-six patients who had undergone upper GI surgery, 38 patients who had eosinophilic esophagitis, one patient who had esophageal stricture owing to an esophageal tumor, two patients who had no available EGD data, and five patients who had not completed the examination were excluded. Additionally, 18 examinations performed during follow-up of the same patient were excluded. A total of 258 patients with upper GI symptoms (258 examinations) with no organic abnormalities on EGD who underwent the first HRM were included (shown in Figure 1).

Evaluation of symptoms

Patients' chief complaints were retrospectively extracted from their medical records. Symptom scores were obtained using questionnaires, such as the FSSG (Frequency Scale for GERD Symptoms) [11], GERD Q [12], reflux symptom index [13], and Eckardt score [14], at the time of HRM. 

HRM

A catheter was inserted nasally, positioned to extend over both the upper and lower esophageal sphincter, and fixed to the nose using tape. After 5 min of rest, 10 mouthfuls of water (5 mL/mouthful) were swallowed while in the supine position. HRM was performed using the Starlet HRM system (Star Medical, Tokyo, Japan). This system has a catheter with a 36-channel solid-state sensor spaced at 1 cm intervals (Unisensor, Attikon, Switzerland). Esophageal motility was described according to CC version 3.0. The upper limit of normal for the integrated relaxation pressure (IRP) was set at 26.1 mmHg, and the normal range for distal contractile integral (DCI) was set at 1,500-13,000 mmHg·s·cm until May 2020 and 1,000-10,000 mmHg·s·cm after June 2020 because of changes in the software version, which also accounted for the differences in IRP and DCI based on the type of HRM catheter used [15,16]. EMDs on HRM were diagnosed using CC v3.0 [4].

Study endpoints

The primary endpoint was the proportion of EMDs in patients with upper GI symptoms. The secondary endpoint was the relationship between upper GI symptoms and the presence of EMDs.

Statistical analysis

A chi-square test was used for intergroup comparisons of qualitative variables. All statistical analyses were performed using IBM SPSS Statistics for Windows, Version 25 (Released 2017; IBM Corp., Armonk, New York, United States). Statistical significance was set at p < 0.05.

Ethical approval

This study was conducted according to the ethical principles of the Declaration of Helsinki for medical research involving human participants. The requirement for informed consent was waived because the data were anonymized during extraction using a correspondence table with numbers unrelated to personal information. The study protocol including waiver of informed consent was approved by the Ethics Committee of Toranomon Hospital, Japan (protocol number: 1625). The information disclosure document of this study was published on our hospital’s website.

## Results

Patient characteristics

Patient characteristics are summarized in Table 1. The mean age was 57 ± 15 years, and 42.4% of patients were male. The most common symptom was dysphagia, observed in 101 patients (39.1%), followed by heartburn in 51 patients (19.8%). Other symptoms listed in Table 1 included nausea and belching, among others. The symptom scores and measured HRM values are shown in Table 1.

**Table 1:** Patient characteristics. FSSG, Frequency Scale for GERD Symptoms; IRP, integrated relaxation pressure; LES, lower esophageal sphincter; DCI, distal contractile integral; DL, distal latency.

Patients, n	258
Age (years), mean ± SD	57 ± 15
Sex (male) n (%)	118 (42.4)
Symptoms	
Dysphagia, n (%)	101 (39.1)
Heartburn, n (%)	51 (19.8)
Chest pain, n (%)	19 (7.4)
Globus sensation, n (%)	36 (14)
Dyspepsia-like, n (%)	29 (11.2)
Others, n (%)	22 (8.5)
Symptom scores	
FSSG, mean ± SD	16.6 ± 8.9
GERD Q, mean ± SD	7.3 ± 2.3
Reflux symptom index, mean ± SD	13.4 ± 9
Eckardt score, mean ± SD	3 ± 3.1
HRM IRP (mmHg), mean ± SD	20.5 ± 10
LES pressure (mmHg), mean ± SD	37.6 ± 35.3
Mean DCI (mmHg·s·cm), mean ± SD	2,959 ± 2,957
Max DCI (mmHg·s·cm), mean ± SD	4,429 ± 4,928
DL, mean ± SD	7.4 ± 1.5

Study endpoints 

Regarding the primary endpoint, the proportion of EMDs based on CC v3.0 in patients with upper GI symptoms was 45.3% (117 of 258 patients) (Table 2). Sixty-nine patients (26.7%) had disorders with esophagogastric junction outflow obstruction (EGJOO), 12 patients (4.7%) had major disorders of peristalsis, and 36 patients (14%) had minor disorders of peristalsis. Among the subcategories of EMDs, EGJOO and ineffective esophageal motility were the most common (36 patients, respectively), followed by achalasia types I-III (33 patients). 

**Table 2:** Disorders of esophageal motility on the Chicago Classification version 3.0 in symptomatic patients. EGJ, esophagogastric junction.

Main categories	Patients, n (%)	Subcategories	Patients, n (%)
Disorders with EGJ outflow obstruction	69 (26.7)	Achalasia types Ⅰ–Ⅲ	33 (12.8)
		EGJ outflow obstruction	36 (14)
Major disorders of peristalsis	12 (4.7)	Distal esophageal spasm	2 (0.8)
		Jackhammer esophagus	4 (1.6)
		Absent contractility	6 (2.4)
Minor disorders of peristalsis	36 (14)	Ineffective esophageal motility	36 (14)
		Fragmented peristalsis	0
Abnormal 117/258 (45.3)			

The results of the secondary endpoint are presented in Table 3. In patients with dysphagia, 66 (65.3%) had EMDs, which was significant (p < 0.001). In contrast, the proportion of EMDs in patients with heartburn or other symptoms was lower (p = 0.011 and 0.026, respectively). Intergroup comparisons of symptom-specific prevalence showed statistically significant differences (p < 0.001), indicating that EMDs were more prevalent among patients with dysphagia.

**Table 3:** Disorders of esophageal motility on the Chicago Classification version 3.0 by symptom. The chi-square test was used. Statistical significance was set at p < 0.05. *Intergroup comparisons of symptom-specific prevalence showed statistically significant differences (p < 0.001)

Symptoms*	Disorders of esophageal motility	Normal	p-value
Dysphagia, n (%)	66 (65.3)	35 (34.7)	<0.001
Heartburn, n (%)	15 (29.4)	36 (70.6)	0.011
Chest pain, n (%)	9 (47.4)	10 (52.6)	0.854
Globus, n (%)	13 (36.1)	23 (63.9)	0.230
Dyspepsia, n (%)	9 (31)	20 (69)	0.100
Others, n (%)	5 (22.7)	17 (77.3)	0.026

Table 4 shows the proportions of each EMD based on CC v3.0 as well as the distribution of various EMD subtypes observed in patients with dysphagia. Among the latter, 45.5% of patients were diagnosed with achalasia type I-III (p < 0.001).

**Table 4:** Proportion of each esophageal motility disorder in the Chicago Classification version 3.0. The Chi-square test was used. Statistical significance was set at p < 0.05. EGJOO, esophagogastric junction outflow obstruction; DES, diffuse esophageal spasm; JE, jackhammer esophagus; AC, absent contractility; IEM, ineffective esophageal motility.

Symptoms	Achalasia types Ⅰ–Ⅲ	EGJOO	DES	JE	AC	IEM	Total	p-value
Dysphagia, n (%)	30 (45.5)	17 (25.8)	2 (3)	4 (6)	2 (3)	11 (16.7)	66	<0.001
Heartburn, n (%)	1 (6.7)	4 (26.6)	0	0	1 (6.7)	9 (60)	15	0.123
Chest pain, n (%)	2 (22.2)	5 (55.6)	0	0	0	2 (22.2)	9	0.654
Globus, n (%)	0	6 (46.2)	0	0	1 (7.6)	6 (46.2)	13	0.198
Dyspepsia, n (%)	0	3 (33.3)	0	0	1 (11.1)	5 (55.6)	9	0.316
Others, n (%)	0	1 (20)	0	0	1 (20)	3 (60)	5	0.343

## Discussion

The present study clarified the proportion of EMDs based on CC v3.0 in patients with upper GI symptoms. The results indicate that 45.3% of patients with upper GI symptoms had EMDs. Additionally, patients with dysphagia are more likely to have EMDs. 

EMDs have been gradually elucidated as HRM has become more widely performed; however, there are few reports on disease frequency in a large number of patients in Japan. Monrroy et al. [17] reported that EMDs were observed in approximately 50% of patients with upper GI symptoms who underwent HRM, and disorders with EGJOO, major disorders of peristalsis, and minor disorders of peristalsis were present in 11.3%, 14%, and 24.5% of patients, respectively. In contrast, they were observed in 26.7%, 4.7%, and 14%, respectively, in the present study. This difference may be due to differences in patient backgrounds. In comparison with the chief complaints of the patients, the previous study [17] included dysphagia in 29%, gastroesophageal reflux disease (GERD) symptoms in 55%, chest pain in 5%, and other reasons in 11%, whereas these were 39.1%, 19.8%, 7.4%, and 33.7%, respectively, in the present study. Thus, we speculate that disorders of EGJOO were more frequent in the present study than in the previous study [17]. Another study involving 201 patients demonstrated that 58.7% of the patients had EMDs based on CC v3.0 [18]. In that study, 71% of the patients with dysphagia had EMDs, which was a higher proportion than that observed for other symptoms. Few studies have reported the prevalence of EMDs in Japan. One previous study from Japan reported that EMDs were found in 58 of 100 patients with dysphagia based on CC version 2.0 [19]. The main results of the present study are consistent with most previous reports.

The analysis of EMDs according to symptoms in the present study showed that patients with dysphagia had significantly more EMDs. In addition, patients with dysphagia had various types of EMDs, including achalasia, EGJOO, and major disorders of peristalsis. EMDs were found in 47.4% of patients with chest pain, although no significant difference was observed because of the small number of patients. These results suggest that symptoms, such as dysphagia and chest pain, may have a stronger relationship with EMDs. Dysphagia has been reported as the primary clinical presentation in both achalasia and EGJOO [20]. In achalasia, common symptoms include dysphagia, regurgitation of ingested food, and chest pain [21]. In this study, one patient with heartburn was diagnosed with achalasia, highlighting that achalasia can sometimes present with symptoms suggestive of GERD. Notably, when GERD symptoms remain inadequately controlled despite optimized lifestyle modifications and pharmacotherapy, further evaluation may be useful, including assessment of esophageal peristaltic function and exclusion of achalasia using HRM [22]. In this study, heartburn and other symptoms were associated with a relatively lower prevalence of EMDs. Symptoms refractory to treatment may more strongly indicate the presence of EMDs, suggesting a higher predictive value for targeted diagnostic testing.

The present study has some limitations. First, this was a retrospective study conducted at a single institution in Japan, which may be subject to certain biases, including selection and information bias. Moreover, chief complaints were extracted from medical records, which may also introduce information bias. Second, a routine esophageal biopsy to exclude eosinophilic esophagitis was not performed in all patients. Third, the information about oral medications, which might affect esophageal motility, is lacking. Fourth, the possible confounding factors such as age, sex were not included in the analysis. Finally, the findings of this study were based on CC v3.0, which may differ from those obtained using CC v4.0. However, few studies have investigated EMDs in Japanese patients presenting with upper GI symptoms, particularly regarding the relationship between EMDs and clinical manifestations. Notably, this study included 258 patients who underwent HRM, presenting a relatively large cohort from a single institution compared with previous reports. We believe that our findings will be useful in daily clinical practice, particularly in guiding the indication for HRM.HRM is an essential and valuable tool for the diagnosis of EMDs. However, it is also a burdensome procedure for patients, and the decision to perform the examination should be guided by the severity and nature of symptoms. Further prospective studies involving larger cohorts are warranted. 

## Conclusions

EMDs were frequently observed in patients with upper GI symptoms based on CC v3.0, especially those with dysphagia. HRM can serve as a valuable diagnostic modality in patients with upper GI symptoms who show no organic abnormalities on EGD. Given the possibility of EMDs, especially achalasia, HRM should be considered as part of clinical practice for patients with dysphagia.
